# Driving personalized medicine forward: the who, what, when, and how of educating the health-care workforce

**DOI:** 10.1002/mgg3.113

**Published:** 2014-09-14

**Authors:** Jeanette J McCarthy

**Affiliations:** Genomic Medicine Initiative, UCSF School of Medicine513 Parnassus Ave HSE901 Box 0794, San Francisco, California, 94143

I spent most of my career as a genome scientist working on advancing personalized medicine in both academic and industry settings. For years now we have heard about the barriers to implementing personalized medicine and each discussion invariably ends with a call to educate health-care providers. The educational challenges are well known and formidable: a crowded curriculum; lack of knowledgeable faculty; lack of evidence-based guidelines; misconceptions about the nature of genomic medicine, and based on my own experience, a lack of enthusiasm by health-care providers to learn about an area that they see as invalidated and expensive (unpublished survey). Many are quick to outline the challenges, but comparatively few have offered viable solutions. Some basic questions remain unanswered: *Who* exactly is it that we need to educate? *What* exactly do they need to know? *When* is the best time in their training or career to be educated? *How* do we move forward, given the real time constraints of doctors in both training and practice?

A couple of years ago, I gave up my research and turned my attention fully to the task of educating health-care providers and other stakeholders in the field of personalized medicine. I have since produced symposia, workshops, and webinars, taught didactic courses in-person and online and launched a consumer magazine on the topic. Through these experiences and monitoring what others have offered, I have developed a framework for personalized medicine education that I'd like to share.

First I will address the *Who* question. The traditional model of genetic testing places medical geneticists and genetic counselors at the center of genetic services. While there remains an important role for these professions, they alone cannot, nor can we afford to have them, absorb the entire workload of genomic medicine. According to the American Board of Genetic Counselors, there are currently ∼3500 certified genetic counselors in the US (www.abgc.net/About_ABGC/GeneticCounselors.asp), approximately one for every 245 actively licensed physicians (www.nationalahec.org/pdfs/fsmbphysiciancensus.pdf). As genomic medicine infiltrates more medical disciplines, the genetics specialty model becomes more difficult to sustain. Instead, it is likely that primary health-care providers will assume prominent roles at the front lines of genomic medicine, taking responsibility for administering new genomic tests and fielding questions from informed patients. As such, primary care physicians, nurses, pharmacists, and other health professionals will require a baseline understanding of genomics to keep pace with medical advances.

So how well prepared are physicians to deliver personalized medicine? In 2013, the NHGRI convened a number of professional organizations at a Genomic Medicine Centers Meeting on Physician Education in Genomics. Most noteworthy was a survey of ∼500 internists in the American College of Physicians (ACP) about their knowledge and skills in genomics (www.youtube.com/watch?v=jJ-ZKIT94sA&list=PL1ay9ko4A8snrWm1tCXVtR5CwBRapSylA&index=9). The survey found that 60% felt that their basic genetic knowledge was adequate, but 25% or less had adequate knowledge about the following: testing and intervention; ethical, legal, and social issues; understanding/interpreting/explaining results; interpreting lab performance; insurance coverage. Furthermore, 25% or less felt they had adequate skills for finding and using practical information about tests, like which tests to use when, and where to find evidence/guidelines on tests.

These data shed light on the *What* question…..what exactly do we need to be teaching physicians in order for them to practice personalized medicine? Basic genomic literacy is necessary to a degree, especially to be able to accurately converse in the language of genome science. But to what degree? According to the ACP survey, most physicians already feel their basic genetic knowledge is adequate. The biggest challenge is distilling an entire field down to just the essentials. One doesn't need to be a mechanic in order to drive a car and similarly, one should not need the equivalent of a graduate degree in genome science in order to practice personalized medicine. The Inter-Society Coordinating Committee (ISCC) for Physician Education in Genomics recently published a framework for the development of genomics practice competencies that attempts to strike this balance (Korf et al. 2014). Moreover, education that is limited to foundational concepts, but lacks practical information (available tests, guidelines for their use, and interpretation of results) is of little use. This is reflected in the ACP survey where less than a quarter of surveyed physicians knew where to find practical information, or how exactly to use it. Even more concerning is that educators themselves may struggle to find good sources of practical information to share.

Is there an equivalent of an owner's manual, a driver's license and Jiffy Lube for physicians to practice personalized medicine? The car analogy is obviously not perfect. We trust that a car we buy and drive is safe. There are well-defined rules of the road, governed by traffic laws. Through practice we learn the common courtesies of pulling over for an ambulance, turning the brights off in oncoming traffic, performing a zipper merge. In contrast, the safety of many genomic tests is questionable and the regulatory landscape of laboratory developed tests still uncertain. Ethical issues that arise may be too important to just pick up during practice. But that shouldn't stop us from developing an owner's manual full of practical information, a licensing process that tests a user's competency, and a place any physician can go to get advice or services that they themselves are uncomfortable rendering.

*When* should we educate health-care providers? Ideally personalized medicine education should start early in training - during fellowships and residency, maybe even earlier. A number of schools and organizations such as the Association of Professors of Human and Medical Genetics (APHMG) are addressing the integration of personalized medicine into the medical school curriculum (Demmer and Waggoner 2014). But presently, there is an entire workforce of existing health-care providers in need of education, and according to the ACP survey, they don't have the time. The vast majority of physicians surveyed were willing to spend at most 4 h learning about genomic medicine. Their preferred learning formats were print/digital and lecture/presentation online and by far the strongest motivating factor for genomics education was continuing medical education (CME).

With this information in hand, we began to address the *How* question. Last spring, my colleagues at UCSF and I launched the first MOOC (massively open online course) for genomic and precision medicine through Coursera (www.coursera.org/course/genomicmedicine). The goal of the course was to provide health-care workers with both practical information and a conceptual foundation from which to evaluate and deliver genomic and precision medicine in the course of clinical care. As such, it was clinically focused and as succinct as possible while covering a wide range of applications including: genetic testing for complex diseases; Mendelian carrier testing and newborn screening; next-generation sequencing for solving diagnostic dilemmas; cancer genomics; and pharmacogenomics. The entire course consisted of seven 1-h videotaped lectures, and the modular format of each allowed learners to skip over information they already knew or was not relevant to their practice. We offered 14.00 AMA PR Category 1 CME credits for completion of both the entire course and the brief weekly assessments.

The stats for our course were on par with others offered through Coursera. Approximately 8000 people enrolled and visited the site during the course. A total of 195 signed up for “Signature Track,” paying $35 each for the opportunity to get a certificate or CME. Extrapolating from our precourse survey, ∼40% of students were from the US, with over 65 other countries represented. About one quarter of students were involved in patient care. The numbers watching the video lectures dropped off each week from a high of ∼5600 to only ∼1700 by the end.

What did we learn from this course? I think it's fair to say that we learned as much in the process of developing it as we did in delivering it. Some key take-aways include:

- The importance of presenting actual clinical scenarios – through role-playing we were able to contextualize some of the ethical and social issues that arise and highlight various situations that physicians may find themselves in, and these vignettes were very well received.
- The need to craft a lecture from a health-care provider point of view and know what questions they would want answered. Surprisingly, coming up with the questions was not as difficult as finding the answers to those questions.
- Professional exposure to personalized medicine coming into the course was very low. Our precourse survey showed that half of the health-care provider students had never ordered a genomic test, had a patient request a genomic test or bring genomic test results to them to interpret. Only 5–15% had done so frequently.
- The value of embedding optional survey questions into the weekly assessments, exploring the intersection between ethics and genetics, allowing us to gain valuable insights for our own research whilst providing a forum for students to engage in these topics.
- In light of the high drop-out, common across all online learning, the need to consider even shorter “micro-learning” units that can be consumed at the convenience of the learner, even at the point of care.

Where do we go from here? In the continued spirit of democratizing education, we are making the videos freely available to others for teaching purposes upon request, as well as offering the course repeatedly through Coursera. The materials are well suited for a ‘flipped classroom’ teaching model where students can watch the videos at their leisure and attend class to engage in hands-on activities and discussions. These activities may include role-playing clinical scenarios, participating in debates, reconstructing guidelines, and evaluating a student's own genetic data. This course could be thought of as the driver handbook, and the class activities the simulator. While we have yet to tackle the other components to round out our automobile analogy, we hope our framework offers a good start to get this field on the road.

## Conflict of Interest

None declared.
